# Glutathione-Mediated Cu(I)/Cu(II) Complexes: Valence-Dependent Effects on Clearance and In Vivo Imaging Application

**DOI:** 10.3390/nano7060132

**Published:** 2017-06-01

**Authors:** Su-Na Yin, Yuanyuan Liu, Chen Zhou, Shengyang Yang

**Affiliations:** 1 College of Chemistry & Chemical Engineering, Yangzhou University, Yangzhou 225002, China; snyin@yzu.edu.cn (S.-N.Y.); yzulyy@hotmail.com (Y.L.); 2 School of Environmental, Physical and Applied Sciences, University of Central Missouri, Warrensburg, MO 64093, USA; zhou@ucmo.edu

**Keywords:** renal clearable, luminescence, copper complexes, biodistribution, PET imaging

## Abstract

Contrast imaging agents need to be cleared in a reasonable time (less than 72 h), so it is quite urgent to understand the structure, biocompatibility, and metabolism features of imaging agents. In this work, luminescent Cu(I)-GSH complex and their derivative oxidized Cu(II)-GSSG complex have been easily synthesized. Through systematically probing the renal clearance and biodistribution of the as-prepared copper complexes, we found that Cu(I)-GSH complex revealed much more efficient renal clearance and remarkably lower liver accumulation than that of their oxidation states, which could be due to strong protein binding of partial forms of Cu(II)-GSSG complex. Besides, we also attempted to incorporate radioactive copper-64 into Cu(I)-GSH complex for the synthesis of radioactive contrast agent. Indeed, the as-prepared radioactive Cu(I)-GSH complex also showed consistent high efficiency renal excretion, allowing them to be potential PET imaging agents in clinical translation.

## 1. Introduction

The development of contrast agents for bioimaging has aroused great interest in optical imaging, biochemistry, and nanomaterials, due to their vital significance in the early diagnosis of various diseases [1]. In order to propel the contrast and enhance the precision of diagnosis, diverse contrast agents, such as dyes and complexes, have been extensively investigated and some of them show promising clinical operation [2,3,4]. More attractively, thanks to the unique electronic, optical, and magnetic properties, nanoparticles have received much attention in the exploration of contrast probes [5,6]. For example, fluorescence nanoparticles (quantum dots (QDs), metal nanoparticles) can be suitably used in optical imaging system, while magnetic nanoparticles (Fe_3_O_4_) can serve as magnetic resonance imaging agents [7]. In contrast to the conventional imaging molecular probes, nanoparticle-based contrast probes demonstrate many advantages, including intensive fluorescence, high paramagnetism, and so on [8,9]. 

Fluorescence, as a low-cost and non-destructive imaging technique, has been widely employed in preclinical investigations, revealing great promise to shift diagnostic and therapeutic modalities to clinical practice [10]. However, because of the adverse effects of quick bioaccumulation in the reticuloendothelial system (RES; e.g., liver and spleen), most fluorescence agents are not compatible to the biotic issues and thus cannot be adopted in human clinical trace [11]. Therefore, the US Food and Drug Administration (FDA) has issued warnings on some of contrast probes, which can often induce severe side effects and even organ dysfunction [12]. For this reason, the fluorescence contrast agents not only need favorable biocompatibility, but also quick clearance without serious accumulation in organs/tissues after imaging. In our recent works, we found that the decomposable luminescent silver and copper nanoparticles were also able to be eliminated via renal system, different from conventional nanoparticles that were mainly accumulated in the RES and other vital organs/tissues [13,14]. These examples are the initial studies that demonstrated degradable nanoparticles into complexes could also be excreted through kidneys, revealing significant understanding of nanotoxicity and dissociation behavior of inorganic nanoparticles. Actually, diverse small molecular fluorescence probes have demonstrated potential translation to clinical applications [15,16,17,18,19]. While important achievements have been attained in the fabrication of renal clearable probes, further understanding of the charge effect of probes in in vivo metabolism was seldom considered.

Copper, the third most abundant trace element in the human body, is essential for the normal growth and development of animals and human, including erythropoiesis, energy production, nerve conduction, enzyme catalysis, and immune system, etc. [20]. Copper deficiency results in anemia, impaired growth, bone abnormalities, frequent risk of infections, abnormal metabolism, and so on [21]. In contrast, excess copper intake in the body can cause oxidative stress, acute toxicity, and diseases (e.g., Wilson disease) [22]. Although copper reveals vital importance in living organisms, it is barely employed in the biological applications except antibacterial and antifouling, etc. [23]. In this work, we employed glutathione (GSH), which is the most predominant tripeptide biothiol in cells, to coordinate copper ions for the synthesis of luminescent Cu(I)-GSH complex. The as-prepared luminescent Cu(I)-GSH complex reacted easily with molecular oxygen and form non-luminescent Cu(II)-glutathione disulfide (Cu(II)-GSSG) complex [24,25,26]. Through systematic investigation of the renal elimination and biodistribution of two different complexes, we found that much more (~40%) of the Cu(I)-GSH complex can be excreted from kidney than Cu(II)-GSSG complex in 24 h, although their compositions are similar. Consequently, the quantitative analysis of Cu(I)-GSH and Cu(II)-GSSG complexes in vivo is fundamentally significant for further understanding the valence-dependent renal clearance difference and designing various other renal clearable materials with low toxicity towards clinical translation.

## 2. Materials and Methods

### 2.1. Materials

Glutathione (GSH) and sodium hydroxide (NaOH) were purchased from Aldrich. Hydrochloric acid (HCl, 12.1 M), cupric chloride anhydrous (CuCl_2_), and ethanol were obtained from Fisher Scientific (275 Aiken Rd, Asheville, NC 28804, USA). Radioactive ^64^CuCl_2_ solution was provided by University of Wisconsin-Madison. Water was deionized with a Milli-Q system (resistivity ≥ 18.2 MΩ·cm) prior to use. Phosphate buffered saline (PBS) buffer was bought from Lonza. All the chemicals are used as received unless specified.

### 2.2. Synthesis of Cu(I)-GSH and Cu(II)-GSSG Complexes

First, 4 mmol of GSH dissolved in 20 mL water was mixed with 20 mL CuCl_2_ (1 mmol) solution to form a cloudy mixture under stirring. After tuning the pH value to ~4 via 5 M NaOH solution, the mixture turned clear with light yellow color and showed red fluorescence, and the Cu(I)-GSH complex was synthesized with total volume of 50 mL after adding extra deionized water (DI) water. The as-prepared Cu(I)-GSH complex solution was stored under room atmospheres for about one week in the closed vials and the solution color changed to deep blue, indicating the Cu(II)-GSSG complex was successfully achieved. 

For animal study, the as-prepared complexes were purified by adding three times volume of ethanol (21,000 g, 1 min) and the precipitates were dried by *N*_2_ purge. After redissolved in phosphate buffered saline (PBS), the complexes were immediately iv-injected into mice.

### 2.3. In Vitro Study of Cu(I)-GSH Complex

The fresh prepared Cu(I)-GSH complex was incubated in PBS solution with 10 wt. % fetal bovine serum (FBS) at 37 °C. To be specific, 200 μL fresh Cu(I)-GSH complex was purified by repeated precipitation with adding ethanol and then redissolved in PBS solution. 

### 2.4. Experimental Animals

As Animal Care and Use Committee required, the animal studies were implemented under their guidelines [27]. The BALB/c mice of 6–8 weeks old (~20 g), were ordered from the standard source and housed in ventilated cages under standard environmental conditions (23 ± 1 °C, 50 ± 5% humidity and a 12/12 h light/dark cycle) with free access to water and standard laboratory diet.

### 2.5. Biodistribution Studies of Cu(I)-GSH and Cu(II)-GSSG Complex

The mice were sacrificed at 24 h (*n* = 3) after iv-injection of Cu(I)-GSH and Cu(II)-GSSG complexes, respectively. The collected organs were weighed and the copper contents were obtained from the ICP-MS (Fisher Scientific, Asheville, NC, USA).

### 2.6. Instrumentation 

A PTI QuantaMasterTM 30 Fluorescence Spectrophotometer (Birmingham, NJ, USA) was employed for collecting fluorescence spectra. Absorption spectra were measured by a Varian 50 Bio UV–Vis spectrophotometer. Digital camera (S8100, Nikon, Tokyo, Japan) was used to capture the photographic images of samples. 

### 2.7. ICP-MS Measurement of Urine, Blood, and Tissues

The collected organs/tissues were weighed and the copper contents were received via the ICP-MS analysis. The organs, tissues, and blood were dissolved in 2 mL 70% nitric acid in screw-capped glass bottles (20 mL) under sonication for over 12 h, followed by evaporating acid. The residue was then washed by 1% HNO_3_ with final constant volume of 10 mL and centrifuged at 4000 rpm for 10 min to remove precipitates. The copper contents of the resultant clear samples were gained by a PerkinElmer-SCIEXELAN 6100 DRC Mass Spectrometry (940 Winter Street, Waltham, MA, USA). In view of the background of copper, the average copper contents in the various organs/tissues from three BALB/c mice were analyzed via the same treatment process and deducted in the calculation of copper biodistribution.

### 2.8. Micro Positron Emission Tomography (PET)-Computed Tomography (CT) Imaging

The used ^64^Cu-doped Cu(I)-GSH for PET imaging was synthesized and purified like the previous process with minor modification of adding radioactive ^64^CuCl_2_. In vivo mouse imaging was carried out on a Siemens Inveon PET-CT multimodality system (Siemens Medical Solutions, Knoxville, TN, USA) with spatial resolution of ~1.5 mm. ^64^Cu-doped Cu(I)-GSH was administered to the BALB/c mouse (6–8 weeks) via iv-injection. PET images were collected immediately post injection (p.i.) for 1 h. The CT projections (360/rotation) were conducted with a power of 80 kV(p), current of 500 μA, exposure time of 145 ms, and binning size of 4. The first 1 h PET images were reconstructed into 20 frames of 180 s using a 3D Ordered Subsets Expectation Maximization (OSEM3D/MAP) algorithm. The CT reconstruction protocol used a down sample factor of 2, was set to interpolate bilinearly, and used a Shepp–Logan filter. The PET and CT images were coregistered in Inveon Acquisition Workplace (Siemens Medical Solutions, Knoxville, TN, USA) for analysis. Circular regions of interest (ROI) were drawn manually, encompassing the kidney and bladder in all planes containing the organs. The target activity was calculated as percentage injected dose per gram (%ID/g), which is defined as (activity (mCi/mL))/(total injected dose (mCi))/density (assuming density = 1 g/mL).

## 3. Results and Discussion

**Figure 1:**
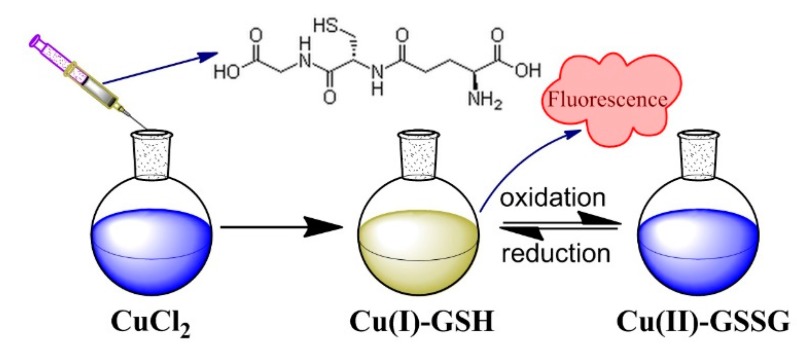
Schematic illustration for the preparation of Cu(I)-GSH and Cu(II)-GSSG complexes.

Figure 1 shows the schematic process for the synthesis of Cu(I)-GSH and Cu(II)-GSSG complexes. Luminescent Cu(I)-GSH was prepared by simply mixing GSH and CuCl_2_, and tuning the pH of the solution to ~4. After being stored under room temperature, the fluorescence of Cu(I)-GSH gradually quenched and the Cu(II)-GSSG complex subsequently took on a deep blue color.

**Figure 2:**
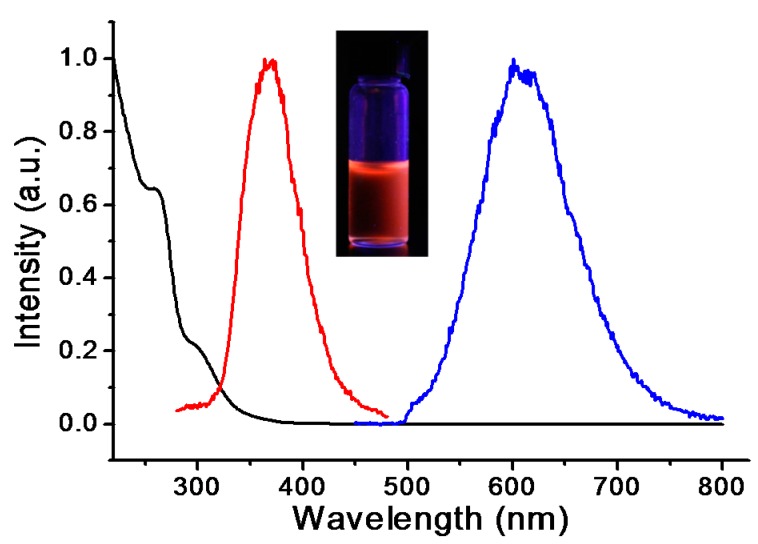
Absorption, excitation, and emission spectra of luminescent Cu(I)-GSH complex. (λ_abs_ = 255 and 300 nm; λ_ex,max_ = 370 nm, λ_em,max_ = 615 nm). Inset: Luminescent Cu(I)-GSH complex under ultraviolet with excitation wavelength of 365 nm. The fluorescence of Cu(I)-GSH complex was conducted in aqueous solution with pH of ~4.

The photophysical characterization of luminescent Cu(I)-GSH was conducted after the purification to remove free ligands and salts. Due to the sulfur-metal coordination, the charge transfer absorbance from ligand to metal can be found in UV–Vis spectroscopy [28]. Indeed, as shown in Figure 2, a typical absorption at ~255 nm can be attributed to the ligand charge transfer band of Cu(I)-GSH complex [29]. The as-prepared Cu(I)-GSH complex showed intensive red fluorescence with the maximum excitation and emission of ~370 and ~615 nm, respectively (Figure 2). This observed fluorescence emission was also due to the ligand–metal charge transfer between GSH and copper [30]. Generally, Cu(I)-thiolate complex does not display luminescence under room atmosphere because of the interaction between solvent and chelation site [31]. However, the luminescence of fresh prepared Cu(I)-GSH complex was kept well under room temperature, indicating the solvent interactions were abolished in our system. 

**Figure 3:**
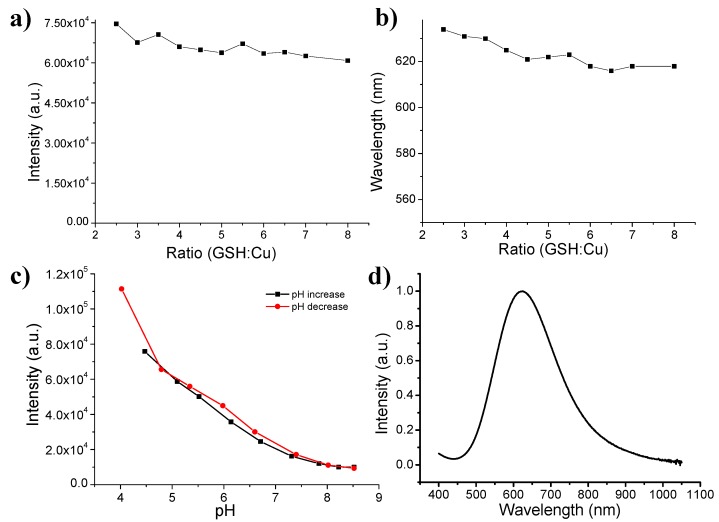
(**a**) Fluorescence intensity and (**b**) emission wavelength of Cu(I)-GSH complex at different *R*_GSH/Cu_; (**c**) Fluorescence intensity of Cu(I)-GSH complex (Cu/GSH = 1:4 mol/mol) at different pH values; (**d**) Absorption spectrum of non-luminescent Cu(II)-GSSG complex.

Next, we examined the ratio of GSH to copper (R_GSH/Cu_) effect on the fluorescence intensity and emission wavelength. It can be found that the fluorescence intensity was almost unvaried when the R_GSH/Cu_ increased from 2.5 to 8 (Figure 3a). Moreover, the emission wavelengths were also close under different R_GSH/Cu_ (Figure 3b). A slight blue shift of fluorescence might be due to the little enhanced ligand–metal charge transfer, which can be further ascribed to the antioxidation when the concentration of GSH was increased. Interestingly, we also found the pH-dependent fluorescence feature of as-obtained Cu(I)-GSH complex. When the pH of solution was gradually increased by adding NaOH solution, the fluorescence intensity of Cu(I)-GSH complex revealed closely-linear decreases (Figure 3c). At low pH, equilibrium favors the presence of protonated GSH with thiol form, while GSH partially shows thiolate form (GS^−^) as the pH is increased [32]. It is well known that thiol form benefits the charge transfer and thus enhances the fluorescence intensity [33]. After the pH was reduced to acidic condition, the fluorescence showed a progressive recovery that is still nearly linear (Figure 3c).

As stated above, Cu(I)-GSH complex will be oxidized and form Cu(II)-GSSG complex, which exhibit non-fluorescence. However, the Cu(II)-GSSG complex shows a typical absorption peak at ~625 nm [34,35]. Consequently, the portion of formed Cu(II)-GSSG complex can be traced by its absorbance spectroscopic properties. When the absorption plateau was reached, the Cu(I)-GSH complex was totally oxidized to become Cu(II)-GSSG complex (Figure 3d), indicating that the reaction was finished and the as-obtained Cu(II)-GSSG complex can be purified for further in vivo study. 

**Figure 4:**
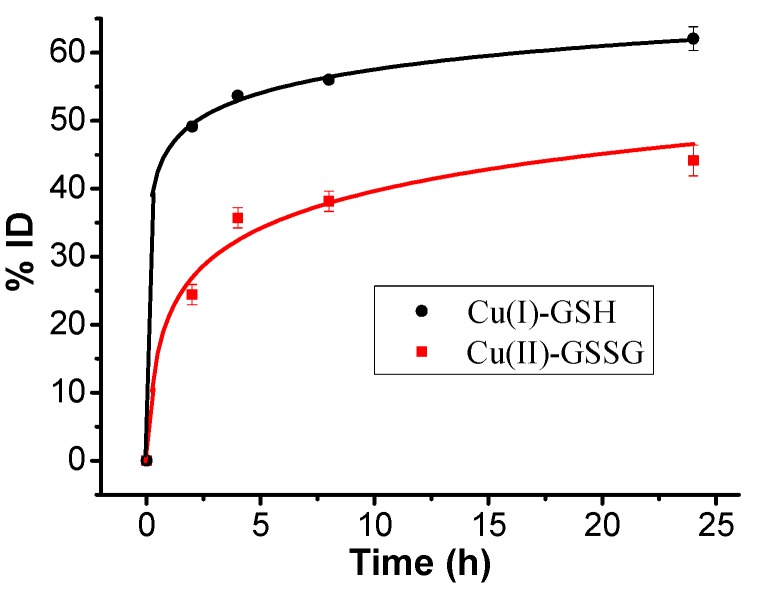
Copper contents in the urine of BALB/c (*n* = 3) at different times post injection (p.i.).

**Figure 5:**
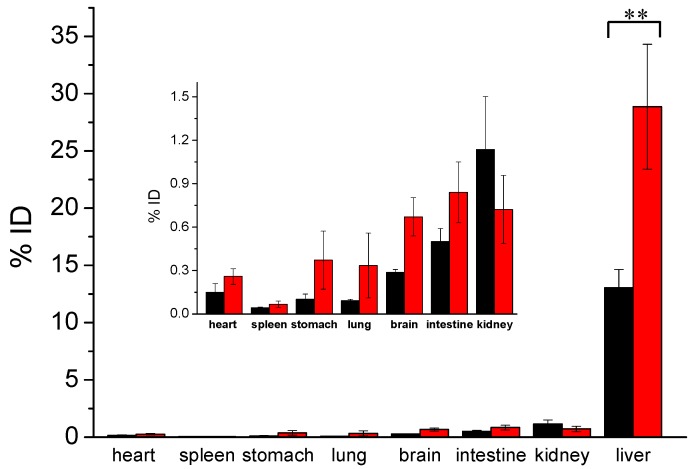
Biodistribution of Cu(I)-GSH and Cu(II)-GSSG complexes in BALB/c mice (*n* = 3) 24 h after the tail-vein injection. (“**”, significance of difference).

To examine the discrepancy of in vivo behavior, Cu(I)-GSH and Cu(II)-GSSG complexes were iv-injected into BALB/c mice, respectively. The urine of the post-injected mice was carefully collected in metabolic cage. Quantitative analysis of copper content by ICP-MS has shown that over 60%ID (injection dose) of Cu(I)-GSH complex was cleared into the urine 24 h p.i. (post injection) and about 50%ID of copper was rapidly excreted from the mice in the first 2 h p.i. (Figure 4), while ~43%ID of Cu(II)-GSSG complex can be eliminated into the urine 24 h p.i., and only ~24%ID was detected at 2 h p.i. (Figure 4). This result indicated a faster clearance of Cu(I)-GSH complex than that of Cu(II)-GSSG complex from the mouse body. Further metabolism analysis was conducted by examining the biodistribution of Cu(I)-GSH and Cu(II)-GSSG complexes. In view of the background of copper, the average copper contents in the various organs/tissues from three BALB/c mice have been deducted. As shown in Figure 5, the Cu(I)-GSH and Cu(II)-GSSG complexes have been mainly excreted from the body except partial fraction accumulated in the liver (~13%ID for Cu(I)-GSH and ~27.5%ID for Cu(II)-GSSG). It should be noted the accumulated copper in the liver of the mice iv-injected with Cu(II)-GSSG complex was about twice higher than the mice iv-injected with Cu(I)-GSH complex; however, no visible discrepancy of copper accumulation in the spleen between two kinds of complexes. This suggested that the biodistribution of copper complexes was remarkably different to CuNPs, which shows size-dependent biodistribution and bigger size usually induces severe accumulation in the spleen. Consequently, the different renal clearance and biodistribution of Cu(I)-GSH and Cu(II)-GSSG complexes might be attributed to their valence discrepancy. Indeed, previous work has confirmed there is complex forms of Cu(II)-GSSG complex with positive valence (e.g., Cu(GSSG)H_3_^+^) [36], which brings in strong protein binding [37] and thus leads to much more accumulation in the liver than Cu(I)-GSH complex [38]. 

**Figure 6:**
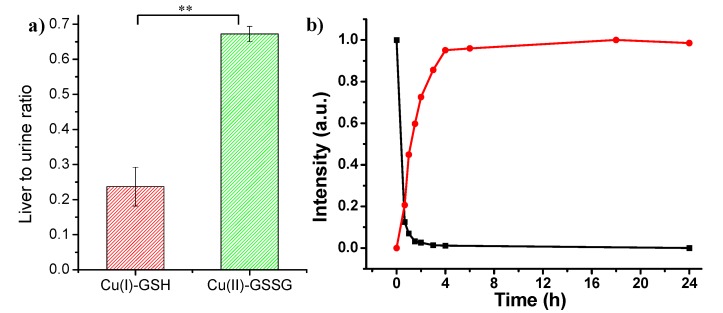
(**a**) Liver-to-urine ratios of Cu(I)-GSH and Cu(II)-GSSG complexes at 24 h p.i.; (**b**) Time-dependent fluorescence intensity of Cu(I)-GSH complex (black curve) and absorption intensity (625 nm) of formed Cu(II)-GSSG complex from the oxidation of Cu(I)-GSH complex (red curve). (“**”, significance of difference).

Further, liver-to-urine ratio (~0.67) of Cu(II)-GSSG complex is about three times higher than that of Cu(I)-GSH complex (Figure 6a). In addition, in vitro incubation study of Cu(I)-GSH complex revealed that Cu(I)-GSH complex was quickly oxidized to Cu(II)-GSSG complex in the first 4 h, which indicated the accumulation of Cu(I)-GSH complex in the liver may be induced by the oxidation of Cu(I)-GSH into Cu(II)-GSSG complex (Figure 6b). 

**Figure 7:**
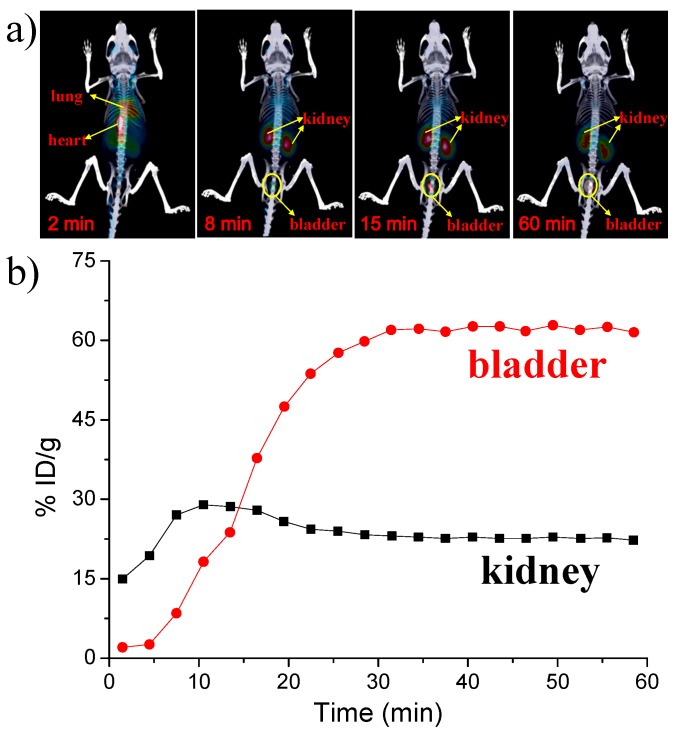
(**a**) Representative microPET-CT images of BALB/c mice at different times after intravenous injection of ^64^Cu-doped Cu(I)-GSH complex; (**b**) Time-dependent kidney and bladder biodistribution of ^64^Cu-doped Cu(I)-GSH complex, the target activity was calculated as percentage injected dose per gram (%ID/g), which is defined as (activity (mCi/mL))/(total injected dose (mCi))/density, (assuming density = 1 g/mL).

Finally, because copper-64 with a half-time of 12.7 h has favorable decay properties (β^+^, 0.653 MeV, 17.8%; β^−^, 0.579 MeV, 38.4%) allows it as an ideal radioisotope for positron emission tomography (PET) imaging, great efforts have been devoted to ^64^Cu-based radiopharmaceuticals for clinical and biomedical research applications. In the current work, we introduced radioactive copper-64 in the synthesis of renal clearable luminescent Cu(I)-GSH complex to obtain radioactive ^64^Cu-doped Cu(I)-GSH complex in vivo PET imaging. The in vivo study of ^64^Cu-doped Cu(I)-GSH complex was explored in BALB/c mice. Consistent with the previous results of Cu(I)-GSH complex in the urine, most of the ^64^Cu-doped Cu(I)-GSH complex directly reached kidney in very short time, followed by going to the bladder. Figure 7 demonstrates time-dependent kidney and bladder biodistribution of ^64^Cu-doped Cu(I)-GSH complex in the first 1 h. Notably, the percentage injected dose per gram (%ID/g) here is a proportion of the injected dose for a voxel. As indicated in Figure 7, the concentration of ^64^Cu-doped Cu(I)-GSH complex in kidney always kept at 15%–30%ID/g, further confirming the prompt renal metabolism of the Cu(I)-GSH complex. Besides the kidney, the intensity in the bladder initially increased sharply and also preserved at high level over 60%ID/g, consistent with the results obtained in the time-dependent urine analysis (Figure 4). Consequently, the as-prepared luminescent Cu(I)-GSH complex can be rapidly cleared to urine, remarkably reducing toxicity of heavy metals, and serving as potential agents for in vivo biomedical applications.

## 4. Conclusions

In summary, we have successfully synthesized luminescent Cu(I)-GSH complex and oxidized non-luminescent Cu(II)-GSSG complex, followed by studying their clearance behavior and in vivo distribution. It has been found that there is obvious difference in renal clearance and liver accumulation of two kinds of copper complexes. Cu(I)-GSH complex showed much higher renal clearance and lower liver accumulation than that of Cu(II)-GSSG complex, which was ascribed to the higher negative charge of Cu(I)-GSH complex being able to facilitate greater clearance than Cu(II)-GSSG complex [39,40]. Moreover, we have demonstrated radioactive Cu(I)-GSH complex can act as a potential PET contrast agent. To our best knowledge, this is the first example to study the valence-dependent clearance difference of copper/glutathione complexes that may serve as a biological imaging agent. Thus, it is meaningful for understanding the toxic metal ions and oxygen species, and metabolism behavior of various promising in vivo agents.

## References

[1] Cheng Z.L., Al Zaki A., Hui J.Z., Muzykantov V.R., Tsourkas A. (2012). Multifunctional nanoparticles: cost versus benefit of adding targeting and imaging capabilities. Science.

[2] Youns M., Hoheisel J.D., Efferth T. (2011). Therapeutic and diagnostic applications of nanoparticles. Curr. Drug Targets.

[3] Ravindran A., Chandran P., Khan S.S. (2013). Biofunctionalized silver nanoparticles: Advances and prospects. Colloids Surf. B Biointerfaces.

[4] Cutler C.S., Hennkens H.M., Sisay N., Huclier-Markai S., Jurisson S.S. (2013). Radiometals for combined imaging and therapy. Chem. Rev..

[5] Gao X.H., Cui Y.Y., Levenson R.M., Chung L.W.K., Nie S.M. (2004). In vivo cancer targeting and imaging with semiconductor quantum dots. Nat. Biotechnol..

[6] Park J.H., Gu L., Maltzahn von G., Ruoslahti E., Bhatia S.N., Sailor M.J. (2009). Biodegradable luminescent porous silicon nanoparticles for in vivo applications. Nat. Mater..

[7] Longmire M., Choyke P.L., Kobayashi H. (2008). Clearance properties of nano-sized particles and molecules as imaging agents: considerations and caveats. Nanomedicine.

[8] Choi S., Dickson R.M., Yu J. (2012). Developing luminescent silver nanodots for biological applications. Chem. Soc. Rev..

[9] Zhang L., Dong S., Nienhaus G.U. (2011). Ultra-small fluorescent metal nanoclusters: Synthesis and biological applications. Nano Today.

[10] Ishizawa T., Fukushima N., Shibahara J., Masuda K., Tamura S., Aoki T., Hasegawa K., Beck Y., Fukayama M., Kokudo N. (2009). Real-time identification of liver cancers by using indocyanine green fluorescent imaging. Cancer.

[11] Nel A., Xia T., Meng H., Wang X., Lin S., Ji Z., Zhang H. (2013). Nanomaterial toxicity testing in the 21st century: Use of a predictive toxicological approach and high-throughput screening. Acc. Chem. Res..

[12] Chen Y., Ye D., Wu M., Chen H., Zhang L., Shi J., Wang L. (2014). Break-up of two-dimensional MnO2 nanosheets promotes ultrasensitive pH-triggered theranostics of cancer. Adv. Mater..

[13] Yang S., Zhou C., Cai X.J. (2015). Glutathione-triggered luminescent silver nanoparticle: A urinary clearable nanoparticle for potential clinical practice. Colloids Surf. B Biointerfaces.

[14] Yang S., Sun S., Zhou C., Hao G.Y., Liu J.B., Ramezani S., Yu M.X., Sun X.K., Zheng J. (2015). Renal clearance and degradation of glutathione-coated copper nanoparticles. Bioconj. Chem..

[15] Hilderbrand S.A., Weissleder R. (2010). Near-infrared fluorescence: Application to in vivo molecular imaging. Curr. Opin. Chem. Biol..

[16] Ntziachristos V., Bremer C., Weissleder R. (2003). Fluorescence imaging with near-infrared light: New technological advances that enable in vivo molecular imaging. Eur. Radiol..

[17] Rao J., Dragulescu-Andrasi A., Yao H. (2007). Fluorescence imaging in vivo: Recent advances. Curr. Opin. Biotechnol..

[18] Weissleder R. (2001). A clearer vision for in vivo imaging. Nat. Biotechnol..

[19] Zhang W.H., Hu X.X., Zhang X.B. (2016). Dye-doped fluorescent silica nanoparticles for live cell and in vivo bioimaging. Nanomaterials.

[20] Bonham M., O’Connor J.M., Hannigan B.M., Strain J.J. (2002). The immune system as a physiological indicator of marginal copper status?. Br. J. Nutr..

[21] Gooneratne S.R., Buckley W.T., Christensen D.A. (1989). Review of copper deficiency and metabolism in ruminants. Can. J. Anim. Sci..

[22] Gaetke L.M., Chow C.K. (2003). Copper toxicity, oxidative stress, and antioxidant nutrients. Toxicology.

[23] Wu C., Zhou Y., Xu M., Han P., Chen L., Chang J., Xiao Y. (2013). Copper-containing mesoporous bioactive glass scaffolds with multifunctional properties of angiogenesis capacity, osteostimulation and antibacterial activity. Biomaterials.

[24] Chen Z., Meng H.A., Xing G.M., Chen C.Y., Zhao Y.L., Jia G.A., Wang T.C., Yuan B.C.H., Wan M.L.J. (2006). Acute toxicological effects of copper nanoparticles in vivo. Toxicol. Lett..

[25] Floriano P.N., Noble C.O., Schoonmaker J.M., Poliakoff E.D., McCarley R.L. (2001). Cu(0) Nanoclusters Derived from Poly(propylene imine) Dendrimer Complexes of Cu(II). J. Am. Chem. Soc..

[26] Speisky H., Lopez-Alarcon C., Olea-Acuna C., Aliaga M.E. (2011). Aliaga ME. Role of superoxide anions in the redox changes affecting the physiologically occurring Cu(I)-glutathione complex. Bioinorg. Chem. Appl..

[27] Pitts M., Bayne K., Anderson L.C., Bernhardt D.B., Greene M., Klemfuss H., Oki G.S.F., Rozmiarek H., Theran P., Van Sluyters R.C. (2002). Institutional Animal Care and Use Committee Guidebook.

[28] Cobine P.A., George G.N., Jones C.E., Wickramasinghe W.A., Solioz M., Dameron C.T. (2002). Copper transfer from the Cu(I) chaperone, CopZ, to the repressor, Zn(II) CopY: Metal coordination environments and protein interactions. Biochemistry.

[29] Battin E.E., Brumaghim J.L. (2008). Metal specificity in DNA damage prevention by sulfur antioxidants. J. Inorg. Biochem..

[30] Dameron C.T., Winge D.R., George G.N., Sansone M., Hu S., Hamer D. (1991). A copper-thiolate polynuclear cluster in the ACE1 transcription factor. Proc. Natl. Acad. Sci. USA.

[31] Yang S., Zhou C., Liu J., Yu M., Zheng J. (2012). One-step interfacial synthesis and assembly of ultrathin luminescent AuNPs/silica membranes. Adv. Mater..

[32] Awad H.M., Boersma M.G., Boeren S., Bladeren van P., Vervoort J.J., Rietjens I.M.C.M. (2002). The regioselectivity of glutathione adduct formation with flavonoid quinone/quinone methides is pH-dependent. Chem. Res. Toxicol..

[33] Casas-Finet J.R., Hu S., Hamer D., Karpel R.L. (1991). Spectroscopic characterization of the copper(I)-thiolate cluster in the DNA-binding domain of yeast ACE1 transcription factor. FEBS Lett..

[34] Postal W.S., Vogel E.J., Young C.M., Greenaway F.T. (1985). The binding of copper(II) and zinc(II) to oxidized glutathione. J. Inorg. Biochem..

[35] Aliaga M.E., López-Alarcóna C., García-Ríob L., Martín-Pastorc M., Speisky H. (2012). Redox-changes associated with the glutathione-dependent ability of the Cu(II)–GSSG complex to generate superoxide Bioorgan. Med. Chem..

[36] Shtyrlin V.G., Zyavkina Y.I., Ilakin V.S., Garipov R.R., Zakharov A.V. (2005). Structure, stability, and ligand exchange of copper(II) complexes with oxidized glutathione. J. Inorg. Biochem..

[37] Yu M.X., Zhou C., Liu J.B., Hankins J.D., Zheng J. (2011). Luminescent gold nanoparticles with pH-dependent membrane adsorption. J. Am. Chem. Soc..

[38] Choi H.S., Ipe B.I., Misra P., Lee J.H., Bawendi M.G., Frangioni J.V. (2009). Tissue-and organ-selective biodistribution of NIR fluorescent quantum dots. Nano Lett..

[39] Cai Z., Anderson C.J. (2014). Chelators for copper radionuclides in positron emission tomography radiopharmaceuticals. J. Label Compd. Radiopharm..

[40] Jones-Wilson T.M., Deal K.A., Anderson C.J., McCarthy D.W., Kovacs Z., Motekaitis R.J., Sherry A.D., Martell A.E., Welch M.J. (1998). The in vivo behavior of per-64-labeled azamacrocyclic complexes. Nucl. Med. Biol..

